# Varicella zoster immune globulin (human) (VARIZIG) in immunocompromised patients: a subgroup analysis for safety and outcomes from a large, expanded-access program

**DOI:** 10.1186/s12879-020-05656-6

**Published:** 2021-01-11

**Authors:** Hayley Gans, Roy F. Chemaly

**Affiliations:** 1 Departments of Pediatrics and Division of Infectious Diseases, Stanford University School of Medicine, 300 Pasteur Drive, Unit G312, Stanford, CA 94305 USA; 2 Department of Infectious Diseases, Infection Control, and Employee Health, The University of Texas MD Anderson Cancer Center, 1515 Holcombe Blvd Unit 402, Houston, TX 77030 USA

**Keywords:** Immunocompromised, Transplant, Varicella, Varicella zoster immune globulin

## Abstract

**Background:**

Immunocompromised children and adults are at increased risk for severe disease and death following varicella zoster virus infection. Varicella zoster immune globulin (human) (VARIZIG) is recommended for post-exposure prophylaxis to prevent or attenuate varicella infection in high-risk individuals.

**Methods:**

An open-label, expanded-access program provided VARIZIG to high-risk individuals exposed to varicella or herpes zoster. Immunocompromised participants were stratified by type of immunocompromising condition (“oncologic immunodeficiency”, “primary immunodeficiency”, “solid organ transplant” [SOT], “hematopoietic cell transplant” [HCT], and “other”). Patient characteristics, type of exposure and varicella outcome, and safety data were assessed.

**Results:**

This analysis included 40 adults (primary [*n* = 6] or oncologic [*n* = 10] immunodeficiencies, history of SOT [*n* = 5] or HCT [*n* = 6], and other [*n* = 13]), and 263 children (primary [*n* = 13] or oncologic [*n* = 152] immunodeficiencies, history of SOT [*n* = 36] or HCT [*n* = 17], and other [*n* = 45]). Among adults and children, 48% vs 72% were exposed to varicella, 38% vs 16% were exposed to herpes zoster, and 15% vs 12% had an unspecified exposure. Overall incidence of varicella infection in adults after VARIZIG use was 6%; incidence of varicella infection in children after VARIZIG use was 7%. Similar rates were noted in each subgroup. Most cases of varicella were mild, with two children developing > 100 lesions and no cases of varicella-related pneumonia or encephalitis. Varicella-related hospitalizations occurred primarily in children with oncologic immunodeficiencies. One serious adverse event (serum sickness) was considered related to VARIZIG and occurred in a child with oncologic immunodeficiency. There were no varicella- or VARIZIG-related deaths.

**Conclusions:**

These data indicate that VARIZIG may reduce severity of varicella in immunocompromised children and adults.

**Trial registration:**

This study was retrospectively registered with the public clinical trial identification NCT00338442 at https://www.clinicaltrials.gov on 20 June 2006.

## Background

Immunocompromised children and adults are at increased risk for severe disease following infection with varicella zoster virus (VZV) [1, 2], including an increased risk of visceral dissemination (i.e. varicella-related pneumonia, encephalitis, hepatitis), secondary bacterial infections, and mortality [2–5]. Even in individuals who previously received two doses of varicella vaccine, an important risk factor is waning immunity as a result of immunosuppressive therapy [6].

Advancements in cancer therapy, the availability of new immunosuppressant medications, and increased rates and survival of organ transplant recipients receiving prolonged immune suppression have resulted in an increased number of immunocompromised adults and children [7, 8]. Varicella vaccination is contraindicated in immunocompromised individuals, including those with malignant conditions and those receiving high-dose systemic immunosuppressive therapy [9]. Passive immunization after exposure with immune globulin specifically targeting VZV has been shown to reduce the severity of varicella infection, and is the generally accepted strategy for prevention in exposed immunocompromised individuals [10–12]. Although there is no official guidance on use of antiviral prophylaxis after varicella exposure, use of antiviral therapy started between 6 and 10 days after exposure and continued for 7 days has been reported to prevent varicella infection after exposure [13].

Varicella zoster immune globulin (human) (VARIZIG, Saol Therapeutics, Roswell, GA, USA) is recommended for post-exposure prophylaxis to prevent or reduce varicella infection in high-risk individuals [9, 12, 14]. VARIZIG is recommended to be administered as soon as possible after varicella or herpes zoster exposure (ideally within 96 h); however, the Centers for Disease Control and Prevention suggests that administration can be as late as 10 days after exposure [12]. An expanded-access program assessed the incidence and severity of varicella infections after post-exposure prophylaxis with VARIZIG in a real-world setting in several high-risk populations, including immunocompromised patients [15]. Although passive immunization is the recommended method for addressing varicella or herpes zoster exposure in immunocompromised individuals, there are limited published data describing varicella incidence and clinical outcomes in patients receiving VARIZIG. As such, we stratified immunocompromised adults and children by type of underlying condition, including primary immunodeficiency, oncology, solid organ transplant (SOT), hematopoietic cell transplant (HCT), and other conditions and analyzed varicella outcome and safety data from the expanded-access program.

## Methods

### Participants

Physician-identified, high-risk individuals who were exposed to varicella or herpes zoster were eligible for inclusion in the study. High-risk participants included immunocompromised children and adults, among others (e.g. preterm infants, in utero–exposed newborns, pregnant women). A full report of the study has been previously published [15]. This analysis focuses on the immunocompromised participants, providing an in-depth analysis of the subgroups within this heterogeneous population. The protocol did not specify what constituted an exposure to varicella or herpes zoster, and was left to the judgment of the investigator. The timing of administration post-exposure was defined based on information provided to the investigator by the participant or family member. Participants were excluded if they had known immunity to varicella, hypersensitivity to blood or blood products, hypersensitivity to any component of VARIZIG, a history of selective immunoglobulin A deficiency, evidence of current varicella or herpes zoster infection at study entry, or evidence of severe thrombocytopenia. Participants with previous varicella immunity who had received an HCT were considered non-immune and could receive VARIZIG per the guidelines established by the Advisory Committee on Immunization Practices [9].

### Study design and treatment

This expanded-access program (NCT00338442) was open-label and took place in a real-world setting at 285 clinical study sites across the United States between March 2006 and April 2013. Study visits occurred at baseline (to determine eligibility, administer VARIZIG, and monitor participants after exposure), between days 1 to 4, between days 7 to 20, and between days 28 to 42. VARIZIG (125 IU/10 kg [up to 625 IU]) was administered once intramuscularly. Ideally, administration occurred as soon as possible after exposure, but administration could occur within 10 days of exposure. This study was conducted in accordance with the Good Clinical Practice Guideline as defined by the International Conference on Harmonisation, the Declaration of Helsinki, and all applicable federal and local regulations and institutional review board guidelines. The protocol and amendments, the informed consent form, and study-related materials were reviewed and approved by a central independent ethics committee (Western Institutional Review Board, Puyallup, WA, USA) before study initiation and throughout the conduct of the study. All patients (or their guardians) provided written informed consent.

### Assessments

#### Assessment of varicella outcome

Patients were assessed for the development of varicella and any varicella-related complications, including but not limited to pulmonary disease and encephalitis. If present, varicella lesions were counted. Varicella was considered complicated if the participant developed more than 100 lesions, pulmonary disease, or encephalitis [16].

#### Safety assessment

Safety was assessed throughout the study, including adverse events (AEs) and serious AEs as defined according to the Medical Dictionary for Regulatory Activities, version 16.0. All AEs were assessed for seriousness, severity, and causality by the investigator.

### Statistical analysis

Immunocompromised participants were grouped by age (children aged 18 years or younger and adults) and then stratified into five groups based on immunocompromising condition: “primary immunodeficiencies”, “oncologic immunodeficiencies”, “history of SOT”, “history of HCT”, and “other” immunodeficiencies. Data were analyzed using descriptive statistics.

## Results

### Participants

There were 40 immunocompromised adults included in the study (Table 1). Of these, 10 individuals (25%) had oncologic immunodeficiencies, 6 (15%) had primary immunodeficiencies, 5 (13%) had a history of SOT, 6 (15%) had a history of HCT, and 13 (32%) had other or unspecified immunocompromising conditions. Most exposures occurred in the household (53%) or the healthcare setting (40%); this pattern held for each subgroup, although the distribution varied to some degree, with SOT recipients more commonly exposed in the hospital (80%), whereas individuals with primary immunodeficiencies were more commonly exposed in the household (83%). There was a relatively equivalent distribution of type of exposure, with 48% exposed to varicella, 38% exposed to herpes zoster, and 15% with an “unknown” or “not specified” exposure in the case report form.

**Table 1:** Baseline demographics and characteristics in immunocompromised adults

Characteristic	Type of immune-compromising condition, *n* (%)					
	All (*n* = 40)	Primary immunodeficiency^a^ (*n* = 6)	Oncologic immunodeficiency^b^ (*n* = 10)	Solid organ transplant^c^ (*n* = 5)	Hematopoietic cell transplant^d^ (*n* = 6)	Other^e^ (*n* = 13)
Age, years						
Mean (SD)	44.3 (17.4)	45.2 (14.6)	43.7 (19.3)	50.8 (15.2)	53.3 (18.2)	37.5 (17.1)
Median (range)	41 (18–75)	48 (29–61)	46 (18–68)	49 (35–71)	61 (24–70)	37 (18–75)
Sex						
Female	19 (48)	3 (50)	4 (40)	2 (40)	2 (33)	8 (62)
Male	21 (53)	3 (50)	6 (60)	3 (60)	4 (67)	5 (38)
Race						
White	25 (63)	4 (67)	7 (70)	5 (100)	2 (33)	7 (54)
Hispanic/Latino	4 (10)	0	2 (20)	0	0	2 (15)
Black/African American	5 (13)	1 (17)	1 (10)	0	2 (33)	1 (8)
Asian	2 (5)	0	0	0	1 (17)	1 (8)
Unknown/Not reported	4 (10)	1 (17)	0	0	1 (17)	2 (15)
Location of VZV exposure						
Household	21 (53)	5 (83)	5 (50)	1 (20)	3 (50)	5 (38)
Healthcare setting	16 (40)	0	4 (40)	4 (80)	3 (50)	7 (54)
School	1 (2)	0	0	0	0	1 (8)
Unknown	2 (5)	1 (17)	1 (10)	0	0	0
Type of VZV exposure						
Varicella	19 (48)	3 (50)	7 (70)	1 (20)	1 (17)	7 (54)
Herpes zoster	15 (38)	2 (33)	1 (10)	4 (80)	4 (67)	4 (31)
Unknown/not specified^f^	6 (15)	1 (17)	2 (20)	0	1 (17)	2 (15)
Timing of VARIZIG administration						
< 96 h after exposure	32 (80)	5 (83)	9 (90)	3 (60)	5 (83)	10 (77)
5–10 days after exposure	8 (20)	1 (17)	1 (10)	2 (40)	1 (16)	3 (23)

Some characteristics may not total 100% because of rounding

^a^Includes participants with primary immunodeficiencies (HIV infection/AIDS [*n* = 2], hypogammaglobulinemia [*n* = 2], immunodeficiency common variable [*n* = 2])

^b^Includes participants with oncologic conditions

^c^Includes participants who have undergone SOT and are taking anti-rejection medication

^d^Includes participants who have undergone HCT

^e^Includes participants with other immunocompromising conditions (adrenoleukodystrophy [*n* = 1], asthma [*n* = 1], aplastic anemia [*n* = 1], chronic renal failure [*n* = 1], immunosuppression [unspecified; *n* = 2], juvenile-onset arthritis [*n* = 1], lupus [*n* = 2], multiple sclerosis [*n* = 1], unknown/unspecified immunodeficiency [*n* = 3])

^f^Participants had known VZV exposure but type of VZV (either varicella zoster or herpes zoster) was not specified or known

There were 263 immunocompromised children included in the study (Table 2). One hundred fifty-two (58%) had oncologic immunodeficiencies, with acute lymphocytic leukemia (*n* = 63) as the most common diagnosis, followed by pre-B acute lymphocytic leukemia (*n* = 13) and neuroblastoma (*n* = 15); all other causes occurred in seven or fewer patients each. There were 36 (14%) children with a history of SOT, 17 (6%) children with a history of HCT, 13 (5%) children with primary immunodeficiencies, and 45 (17%) children with other causes of immunodeficiency. Exposure to varicella or herpes zoster most commonly occurred in the healthcare setting (39%), the household (33%), and school/daycare (21%). Seventy-two percent of children were exposed to varicella, with 16% exposed to herpes zoster and 12% unknown/not specified exposure noted in the case report form.

**Table 2:** Baseline demographics and characteristics in immunocompromised children

Characteristic	Type of immune-compromising condition, *n* (%)					
	All (*n* = 263)	Primary immunodeficiency^a^ (*n* = 13)	Oncologic immunodeficiency^b^ (*n* = 152)	Solid organ transplant^c^ (*n* = 36)	Hematopoietic cell transplant^d^ (*n* = 17)	Other^e^ (*n* = 45)
Age, years						
Mean (SD)	6.4 (4.6)	7.8 (5.3)	6.0 (4.4)	7.2 (4.4)	5.9 (3.7)	6.8 (5.6)
Median (range)	6 (0–17)	6 (0.83–17)	5 (0.25–17)	6 (0.5–16)	6 (1–14)	7 (0–17)
Sex						
Female	125 (48)	4 (21)	78 (51)	15 (42)	6 (35)	22 (49)
Male	138 (52)	9 (69)	74 (49)	21 (58)	11 (65)	23 (51)
Race						
White	163 (62)	8 (62)	91 (60)	26 (72)	10 (59)	28 (62)
Hispanic/Latino	47 (18)	0	34 (22)	5 (14)	3 (18)	5 (11)
Black/African American	32 (12)	4 (31)	18 (12)	2 (6)	2 (12)	6 (13)
Asian	5 (2)	0	3 (2)	0	0	2 (4)
American Indian/Alaskan native	2 (1)	0	1 (1)	0	1 (6)	0
Unknown/Not reported	14 (5)	1 (8)	5 (3)	3 (8)	1 (6)	4 (9)
Location of VZV exposure						
Household	87 (33)	8 (62)	41 (27)	17 (47)	8 (47)	13 (29)
Healthcare setting	102 (39)	2 (15)	75 (49)	8 (22)	5 (29)	13 (29)
School/daycare/playgroup	55 (21)	1 (8)	25 (16)	11 (31)	2 (12)	16 (36)
Other^f^	12 (5)	2 (15)	8 (5)	0	1 (6)	1 (2)
Unknown	6 (2)	0	3 (2)	0	1 (6)	2 (4)
Type of VZV exposure						
Varicella	190 (72)	9 (69)	109 (72)	30 (83)	10 (59)	32 (71)
Herpes zoster	41 (16)	1 (8)	24 (16)	5 (14)	4 (24)	7 (16)
Unknown/not specified^g^	32 (12)	3 (23)	19 (13)	1 (3)	3 (18)	6 (13)
Timing of VARIZIG administration						
< 96 h after exposure	237 (90)	11 (85)	136 (89)	34 (94)	16 (94)	40 (89)
5–10 days after exposure	25 (10)	2 (15)	15 (10)	2 (6)	1 (6)	5 (11)
Unknown	1 (0.4)	0	1 (1)	0	0	0

Some characteristics may not total 100% because of rounding

^a^Includes participants with primary immunodeficiencies (cell-mediated immune deficiency [*n* = 1], combined immunodeficiency [*n* = 2], DiGeorge syndrome [*n* = 2], HIV infection/AIDS [*n* = 2], immunodeficiency common variable [*n* = 3], neutropenia [*n* = 1], tumor necrosis factor receptor–associated periodic syndrome [*n* = 1], and Wiskott-Aldrich syndrome [*n* = 1])

^b^Includes participants with oncologic conditions

^c^Includes participants who have undergone SOT and are taking anti-rejection medication

^d^Includes participants who have undergone HCT

^e^Includes participants with other immunocompromising conditions (adrenoleukodystrophy [*n* = 1], aplastic anemia [*n* = 3], anemia [*n* = 2], aplasia pure red cell [*n* = 1], arthritis [*n* = 1], asthma [*n* = 2], cardiac operation [*n* = 1], chronic granulomatous disease [*n* = 1], Cushingoid [*n* = 1], Evan’s syndrome [*n* = 1], focal segmental glomerulosclerosis [*n* = 1], Goodpasture’s syndrome [*n* = 1], Henoch-Schönlein purpura nephritis [*n* = 1], hypoplastic left heart syndrome [*n* = 1], juvenile-onset arthritis [*n* = 4], McKusick-Kaufman syndrome [*n* = 1], lupus nephritis [*n* = 1], nephrotic syndrome [*n* = 4], ornithine transcarbamylase deficiency [*n* = 1], polyarteritis nodosa [*n* = 1], premature baby [*n* = 7], sarcoidosis [*n* = 1], thrombocytopenia [*n* = 1], ulcerative colitis [*n* = 1], uveitis [*n* = 1], velocardiofacial syndrome [*n* = 1], and unknown/unspecified [*n* = 3])

^f^Other locations of exposure included camp or sporting events

^g^Participants had known VZV exposure but type of VZV (either varicella zoster or herpes zoster) was not specified or known

### Varicella outcomes

**Fig. 1:**
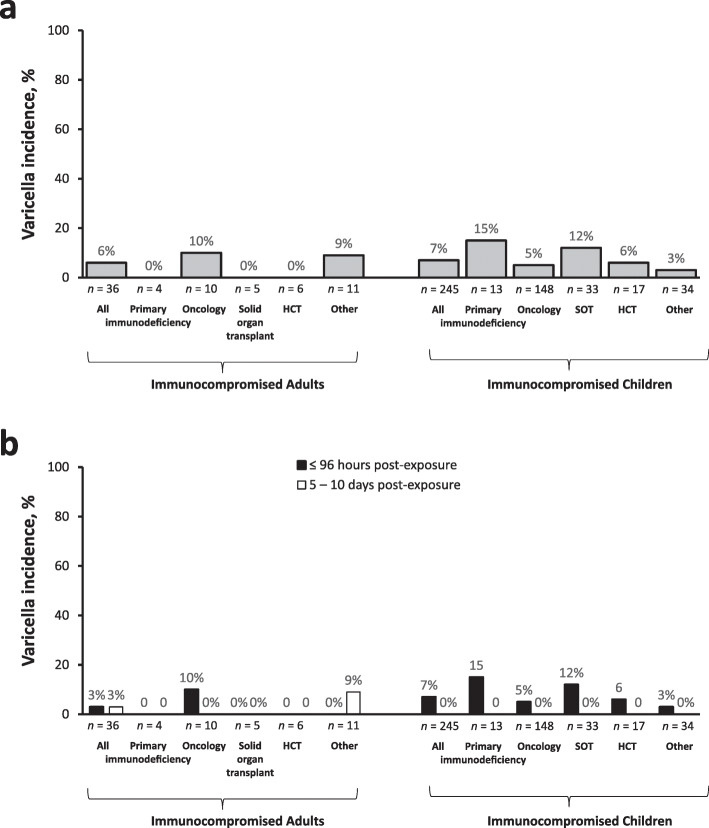
Incidence of varicella in immunocompromised participants. **a** Incidence in immunocompromised participants and subgroups and (**b**) incidence by timing of administration of VARIZIG. Percentage was calculated based on the number of participants with varicella outcome data (noted below the graph)

Of the immunocompromised adults, 80% received VARIZIG within 96 h of varicella or herpes zoster exposure (Table 1). Across subgroups, this pattern was similar, although in adult SOT patients, 60% received VARIZIG in the first 96 h. Overall, immunocompromised adults had a 6% incidence of varicella (Fig. 1a). Within subgroups, adults with oncologic immunodeficiencies had a 10% incidence of varicella and adults with other immunodeficiencies had a 9% incidence of varicella. No patients with primary immunodeficiencies, history of SOT, or history of developed varicella. Varicella outcome data were not available for four adults (two were in the primary immunodeficiency subgroup and two were in the other immunodeficiencies subgroup); these patients were excluded from incidence calculations. Timing of administration did not greatly impact the varicella incidence in adults (Fig. 1b). There were no varicella-related complications (> 100 lesions, pneumonia, or encephalitis) or hospitalizations in immunocompromised adults. Overall, four adults (10%) were treated prophylactically with antiviral therapy, with one patient in the oncologic immunodeficiency subgroup and three patients in the HCT subgroup; none of these patients developed varicella. Antiviral dosing regimens varied, with two patients dosed within the first 2 days of varicella or herpes zoster exposure for a duration of 3 weeks, and two patients were given antiviral therapy 7 days after varicella or herpes zoster exposure for unknown duration (i.e. treatment was still ongoing at study completion).

Ninety percent of immunocompromised children were administered VARIZIG within 96 h of varicella or herpes zoster exposure, this pattern was similar in all subgroups (Table 2). Overall, immunocompromised children had a 7% incidence of varicella (Fig. 1a). This incidence was similar in the subgroup of children with oncologic immunodeficiencies (5%), and children with a history of HCT (6%). Varicella incidence was greater in children with primary immunodeficiencies (15%) and children with a history of SOT (12%). Children with “other” immunocompromising conditions had a 3% incidence of varicella. Varicella outcome data were not available for 18 children (oncologic immunodeficiency, *n* = 4; SOT, *n* = 3; other, *n* = 11); these children were excluded from incidence calculations. When assessed by timing of VARIZIG administration, all cases of varicella occurred in patients who were administered VARIZIG within 96 h of varicella or herpes zoster exposure. Varicella infections were mild, with only two patients developing more than 100 lesions, and no cases of varicella-related pulmonary disease or encephalitis occurred. Most children with oncologic immunodeficiencies who developed varicella (6 of 8 children) and one child with history of SOT were hospitalized for varicella treatment; there were no varicella-related hospitalizations in children with primary immunodeficiencies, history of HCT, or children with other immunodeficiencies. Overall, 49 children (19%) were treated prophylactically with antiviral therapy, of whom 2 were in the primary immunodeficiency subgroup, 32 in the oncologic immunodeficiency group, 6 in the SOT group, 4 in the HCT group, and 5 in the “other” subgroup; none of these patients developed varicella. Antiviral therapy (acyclovir or valacyclovir) was most often dosed (orally or intravenously) in two different regimens: patients were either given antiviral therapy immediately (within 2 days) after exposure for a duration of 2 days to > 1 month, or patients were given antiviral therapy approximately 6 to 10 days after exposure, for a duration of 2 to 3 weeks. A small group of patients were already on antiviral therapy for underlying medical conditions. If patients who received prophylactic antiviral therapy are excluded from the analysis, varicella incidence is not markedly changed (overall pediatric population: 7.5% [16 of 214 participants], oncologic: 6.7% [8 of 120 participants], history of HCT: 7.7% [1 of 13 participants], SOT: 13.3% [4 of 30 participants], primary immunodeficiencies: 18.2% [2 of 11 participants], and other immunodeficiencies: 2.5% [1 of 40 participants].

### Safety

Overall, 15 immunocompromised adults (38%) experienced 51 AEs, of which only one AE of nausea was considered related to VARIZIG (Table 3). Four adults (10%) experienced 10 serious AEs, none of which were considered related to VARIZIG. There was one death (respiratory failure in a patient with AIDS) in the immunocompromised adult group; this death was considered unrelated to varicella or VARIZIG.

**Table 3:** Safety in immunocompromised adults

Preferred term	Type of immune-compromising condition, *n* (%)					
	All (*n* = 40)	Primary immunodeficiency^a^ (*n* = 6)	Oncologic immunodeficiency^b^ (*n* = 10)	Solid organ transplant^c^ (*n* = 5)	Hematopoietic cell transplant^d^ (*n* = 6)	Other^e^ (*n* = 13)
AEs						
Participants, *n* (%)	15 (38)	3 (50)	2 (20)	1 (20)	3 (50)	6 (46)
Total AEs, *n*	51	5	3	1	29	13
Total related AEs, *n*	1	0	1	0	0	0
All related AEs, *n* (%)						
Nausea	1 (3)	0	1 (10)	0	0	0
Serious AEs						
Participants, *n* (%)	4 (10)	1 (17)	1 (10)	0	0	2 (15)
Total serious AEs, *n*	10	1	2	0	0	7
Total related serious AEs, *n*	0	0	0	0	0	0
Deaths, *n* (%)^f^	1 (3)	1 (17)	0	0	0	0

^a^Includes participants with primary immunodeficiencies (e.g. HIV infection/AIDS, hypogammaglobulinemia, immunodeficiency common variable)

^b^Includes participants with oncologic immunodeficiencies

^c^Includes participants who have undergone SOT and are taking anti-rejection medication

^d^Includes participants who have undergone HCT

^e^Includes participants with other immunocompromising conditions (e.g. multiple sclerosis, lupus nephritis, adrenoleukodystrophy, chronic renal failure, asthma, juvenile-onset arthritis)

^f^Death was due to respiratory failure in a woman with AIDS; death was considered unrelated to varicella or VARIZIG

In the group of immunocompromised children, 90 (34%) experienced 402 AEs, of which 53 (13%) were considered related to VARIZIG (Table 4). The most frequently occurring (in more than one participant) AEs that were considered related to VARIZIG were injection site pain (*n* = 5, 2%), headache (*n* = 2, 0.8%), and diarrhea (*n* = 2, 0.8%). There were 42 children (16%) who experienced 93 serious AEs, of which one (serum sickness) was considered related to VARIZIG. There were five deaths: three (caused by acute myeloid leukemia, intracranial hemorrhage, and neuroblastoma) occurred in the oncologic immunodeficiency group, one (caused by acute lymphoblastic leukemia complicated by pneumonia infection) occurred in the group with a history of HCT, and one (caused by congestive cardiac failure) occurred in the “other” immunocompromised group. None of these deaths were considered by the investigators to be related to varicella or VARIZIG.

**Table 4:** Safety in immunocompromised children

Preferred term	Type of immune-compromising condition, *n* (%)					
	All (*n* = 263)	Primary immunodeficiency^a^ (*n* = 13)	Oncologic immunodeficiency^b^ (*n* = 152)	Solid organ transplant^c^ (*n* = 36)	Hematopoietic cell transplant^d^ (*n* = 17)	Other^e^ (*n* = 45)
AEs						
Participants, *n* (%)	90 (34)	5 (38)	62 (41)	9 (25)	4 (24)	10 (22)
Total AEs, *n*	402	15	318	22	30	17
Total related AEs, *n*	53	7	32^f^	6	4	4
Related AEs occurring in > 1 participant^g^, *n* (%)						
Injection site pain	5 (2)	2 (15)	2 (1)	1 (6)	0	0
Headache	2 (0.8)	1 (8)	0	0	1 (6)	0
Diarrhea	2 (0.8)	0	0	1 (6)	1 (6)	0
Serious AEs						
Participants, *n* (%)	42 (16)	3 (23)	28 (18)	5 (14)	2 (12)	4 (9)
Total serious AEs, *n*	93	6	68	5	7	7
Total related serious AEs, *n*	1	0	1	0	0	0
All related serious AEs, *n* (%)						
Serum sickness	1 (0.4)	0	1 (0.6)	0	0	0
Deaths, *n* (%)^h^	5 (2)	0	3 (2)	0	1 (6)	1 (3)

^a^Includes participants with primary immunodeficiencies (e.g. cell-mediated immune deficiency, combined immunodeficiency, DiGeorge syndrome, histiocytosis, HIV infection/AIDS, immunodeficiency common variable, lymphoproliferative disorder, myelodysplastic syndrome, ulcerative colitis, Wiskott-Aldrich syndrome)

^b^Includes participants with oncologic immunodeficiencies

^c^Includes participants who have undergone SOT and are taking anti-rejection medication

^d^Includes participants who have undergone HCT

^e^Includes participants with other immunocompromising conditions (e.g. adrenoleukodystrophy, anemia, aplasia pure red cell, asthma, chronic granulomatous disease, Evan’s syndrome, focal segmental glomerulosclerosis, Goodpasture’s syndrome, Henoch-Schönlein purpura nephritis, hypoplastic left heart syndrome, juvenile-onset arthritis, McKusick-Kaufman syndrome, lupus nephritis, nephrotic syndrome, ornithine transcarbamylase deficiency, polyarteritis nodosa, premature baby, thrombocytopenia, uveitis)

^f^One participant experienced 26 AEs, all of which were considered “conditionally” related to treatment, in that it is unclear how the participant’s underlying condition and treatment for that condition could have been involved in the causality of the reported AEs

^g^Related AEs occurring in one person each included: abdominal pain, arthritis reactive, aspartate aminotransferase, aspartate aminotransferase increased, blood lactate dehydrogenase, cough, fatigue, hemoglobin decreased, hyperglycemia, hypocalcemia, hypomagnesemia, hyponatremia, injection site hematoma, injection site swelling, insomnia, malaise, platelet count decreased, pyrexia, rash erythematous, serum sickness, urticaria, white blood cell count decreased

^h^Deaths were due to acute myeloid leukemia, cardiac failure congestive, intracranial hemorrhage, neuroblastoma, and renal failure

## Discussion

Immunocompromised individuals are more susceptible to severe varicella-related complications, accounting for more than 90% of varicella-related hospital admissions [17]. With increasing numbers of immunocompromised individuals [7, 8] who cannot be vaccinated [9] or lose vaccine-induced immunity due to immunosuppressive therapies [18], there is an ever-present need for protection after varicella or herpes zoster exposure, with passive immunization providing a safe and efficacious strategy in other populations. However, there are limited clinical data published on the use of VARIZIG and the impact on varicella-related outcomes. This analysis reports data from an expanded-access program in subgroups of adult and pediatric immunocompromised patients.

The overall incidence of varicella was 6% in adult immunocompromised patients, and 7% in pediatric immunocompromised patients. Similar incidences occurred in subgroups of patients stratified by type of immunocompromising condition. These incidences are similar to those reported historically in immunocompromised patients. In a 24-year retrospective study of 5777 pediatric patients with cancer, the incidence of varicella was 5% (just 45 of these patients received immunoglobulin prophylaxis) [10]. In 93 adults with multiple myeloma who were receiving lenalidomide, 10 (10.7%) developed varicella infection, and in 132 patients with multiple myeloma who had undergone allogenic HCT, 10 (7.6%) developed varicella infection [1].

Although similar incidences of varicella were reported compared with historical data, one of the benefits of passive immunization lies in its ability to attenuate disease severity in immunocompromised individuals. In immunocompromised participants in this expanded-access program, most cases of varicella were mild; just two children developed varicella with more than 100 lesions, there were no cases of varicella-related complications, and there were no varicella-related deaths. In a pre-vaccination era study that monitored varicella cases (*N* = 77) in immunocompromised children over 11 years, the rate of visceral dissemination was 32%, with mortality occurring in 7% of patients [3]. In the 24-year retrospective study cited above, varicella-related pneumonia occurred in 28% of patients with varicella, with an overall mortality rate of 7% [10]. Another study in immunocompromised children (both oncologic and other causes) reported a 14% varicella-related mortality rate [19]. Retrospective chart reviews of immunocompromised children reported rates of visceral dissemination as high as 21% [20] and 48% [21], with a 14% [20] mortality rate. Data from the US Mortality Multiple Cause of Death public use records (from the National Center for Health Statistics) indicate a total of 155 suspected varicella deaths from 1996 to 2013 from 34 states; of the 77 deaths in which immune status was known, 24 deaths (29%) occurred in immunocompromised persons [22]. Half of the reported deaths occurred in patients with immunocompromising conditions and the other half were in patients being treated with an immunocompromising medication [22]. From 1999 to 2007, deaths occurred in a greater number of involved immunocompromised patients (29%) than deaths reported for immunocompetent individuals (11–18%) [22]. Although vaccination has been shown to reduce the incidence of complications and varicella-related hospitalizations overall, a German varicella surveillance project noted significantly more varicella-related complications in immunocompromised patients, including hematologic complications and systemic bacterial infections [23]. Even with vaccination becoming common practice in many countries, a systematic review of breakthrough varicella noted severe breakthrough disease in healthy and immunocompromised children [24]. Because of bone marrow transplant, previously vaccinated oncology patients may be rendered non-immune [18], therefore increasing their risk for severe varicella infection.

In immunocompromised adults, there was a similar incidence of varicella when comparing the timing of VARIZIG administration post-exposure (3% incidence with administration within and after 96 h post-exposure), much like the pattern reported in the overall study population [15]. However, we report here that in immunocompromised children, all cases of varicella occurred in patients who were administered VARIZIG within 96 h of exposure to varicella or herpes zoster. This may represent a more significant or known varicella or herpes zoster exposure that prompted more aggressive access to care; however, it should be noted that 90% of immunocompromised children were administered VARIZIG within 96 h of exposure.

The results from this analysis demonstrate that VARIZIG is well tolerated and safe in immunocompromised adults and children, confirming previously published, positive safety findings in high-risk patient populations [15]. The proportion of immunocompromised adult and pediatric patients who experienced any AE was similar (38 and 34%, respectively), as well as the proportion of patients with AEs and serious AEs that were considered related to VARIZIG. Most commonly occurring AEs deemed related to VARIZIG are expected adverse drug reactions for immune globulin products (e.g. injection site pain) and/or could be exacerbated by medications used to treat the patient’s underlying condition (e.g. headache, nausea, and vomiting).

This study was limited by the expanded-access, open-label, uncontrolled study design. Some of the limitations of the study did not always identify the type of exposure (e.g. varicella vs herpes zoster). In addition, varicella outcome data are missing for some participants, either because they withdrew early or were lost to follow-up before varicella infection could have developed. In this real-world setting, each physician determined whether the exposure qualified as needing prophylaxis and it is unclear if all cases would have resulted in the development of varicella; as such, these data need to be interpreted carefully. In addition, several participants received VARIZIG and antiviral therapy targeted against varicella or other viral infections; thus, the role of each of these interventions in varicella prevention in these individuals cannot be determined.

In conclusion, these data indicate that passive immunization with VARIZIG may prevent and/or reduce disease severity in immunocompromised children and adults, supporting its use for disease prevention in exposed high-risk individuals. Clinicians should be aware that patients who previously received two doses of varicella vaccine may no longer be immune after immunosuppressive therapies or after undergoing SOT or HCT [6]; as a result, previous immunity to varicella through vaccination should not always be a factor when considering treatment with VARIZIG if patients have undergone immunosuppressive treatment.

## Data Availability

The data underlying this study belong to Saol Therapeutics. Interested researchers can send data access requests to info@saolrx.com.

## Abbreviations

AE: Adverse event
HCT: Hematopoietic cell transplant
SOT: Solid organ transplant
VARIZIG: Varicella zoster immune globulin (human)
VZV: Varicella zoster virus
